# Hyperlipidemic Abdominal Crisis: A Unique Presentation of Isolated Severe Hypertriglyceridemia

**DOI:** 10.7759/cureus.77503

**Published:** 2025-01-15

**Authors:** Michael Mohseni, Ayelet Elwaya, Alex Lu

**Affiliations:** 1 Emergency Medicine, Mayo Clinic, Jacksonville, USA

**Keywords:** emergency medicine, hyperlipidemic abdominal crisis, insulin drip, pancreatitis, plasmapheresis, severe hypertriglyceridemia

## Abstract

Severe hypertriglyceridemia, defined as levels >1000 mg/dL, is a condition that manifests in various ways including hyperlipidemic abdominal crisis (HLAC) with risk of progression to pancreatitis. We discuss the unique presentation of HLAC in a 50-year-old patient whose diagnosis remained unclear on initial presentation. Initial laboratory evaluation and imaging were unrevealing, but laboratory personnel relayed concerns for lipemic blood samples, prompting a lipid panel in the Emergency Department (ED). Triglycerides measured 2562 mg/dL helping to confirm probable diagnosis of HLAC. After consultation with Endocrinology, the patient was admitted for an insulin infusion with glucose-containing maintenance fluids to help treat the very severe hypertriglyceridemia. The patient was discharged in good condition on hospital day 3 with triglyceride levels down to 405 mg/dL and prescribed a new oral agent, Icosapent ethyl at 2 g orally daily. Clinicians evaluating patients for acute abdominal complaints should be alert to the possible diagnosis of HLAC given the potential for deterioration to pancreatitis and concomitant complications. Prompt management with an insulin infusion or plasmapheresis along with appropriate consultation with specialists is crucial to mitigate the risk of adverse outcomes in this rare abdominal emergency.

## Introduction

Hypertriglyceridemia is defined as elevated fasting serum levels of triglycerides greater than 150 mg/dL. The Adult Treatment Panel III report of the National Cholesterol Education Program (NCEP ATP III) developed three additional subcategories to determine the severity of hypertriglyceridemia as follows: borderline high triglycerides 150-199 mg/dL, high triglycerides 200-499 mg/dL, and very high triglycerides greater than 500 mg/dL [1]. A 2002 NCEP ATP III revision added additional categories of severe hypertriglyceridemia, defined as fasting serum triglycerides levels of 1000-1999 mg/dL, and very severe hypertriglyceridemia for levels greater than 2000 to account for complications not usually seen in patients with moderate to high levels [1]. 

While hypertriglyceridemia occurs in almost a quarter of the United States population, severe and very severe hypertriglyceridemia occurs only in roughly one in 5000 individuals (less than 1% of the general population) [2]. Changes to hypertriglyceridemia subcategories and classification of risk factors were made to assist with guidelines for diagnosis and management. Elevated triglyceride levels can be observed in asymptomatic patients as part of risk factor screening, and the main management goal is to focus on atherosclerotic cardiovascular disease secondary prevention with secondary risk factor reduction [1,2]. Patients with severe and very severe elevated triglycerides, however, face a higher incidence of symptomatic hypertriglyceridemia and subsequent complications including acute pancreatitis, chylomicronemia syndrome, and HLAC, as seen in our patient’s case [2]. HLAC is defined as “pancreatitis-like” abdominal pain usually associated with nausea and vomiting; however, unlike acute pancreatitis, serum lipase and amylase levels may be within normal limits and abdominal imaging does not necessarily show pancreatic changes [3].

Providers in the ED must maintain a high level of suspicion for the diagnosis of severe and very severe hypertriglyceridemia given that the lipid panel is not routinely a part of abdominal pain evaluation in the acute setting. Additionally, triglycerides are best measured when fasting, which is often not the case with ED patients. When laboratory testing (such as the complete blood count, complete metabolic panel, or liver function panel) appears opalescent to milky with the inability to accurately report results, this may indicate concern for lipemia and the need for further investigation of the patient's lipid panel [2].

Patients with HLAC typically have a good prognosis with early diagnosis and intervention. Treatment is usually guided by the acuity of the condition, with rapid lowering of triglyceride levels to under 500 ml/dL as the primary goal [4]. Plasmapheresis and insulin infusion are the two main modalities of acute treatment along with cessation of exacerbating secondary factors and initiation of oral medication therapy [4]. Delay in treatment may lead to complications such as repeat abdominal crisis episodes, progression to pancreatitis, and multi-organ failure [4,5].

Here, we describe a case that is one of few reported in the literature of HLAC, presenting solely as abdominal discomfort that was not associated with pancreatitis, highlighting the importance of a high degree of suspicion to effectively diagnose a patient with severe or very severe hypertriglyceridemia in the ED setting.

## Case presentation

A 50-year-old man presented to the Emergency Department (ED) with progressive abdominal discomfort over the previous four weeks. Although he described the abdominal discomfort as generalized, the sensation of pain was most pronounced in the mid to upper abdomen. The discomfort was associated with clear, morning-time emesis on several occasions. He did not report any dark or bloody stools but did report an increase in the frequency of stools to approximately four to six bowel movements per day. The remainder of the review of systems was unremarkable other than a report of moderate fatigue. There was no history of abdominal surgeries. Past medical history was significant for generalized anxiety disorder, hyperlipidemia, hypertension, hepatic steatosis, and alcohol consumption of one to two drinks daily. He had visited his primary care provider approximately three weeks prior to the ED encounter for the same symptoms; at that juncture, he was prescribed omeprazole 40 mg daily and his escitalopram was adjusted from 10 mg daily to 15 mg daily. Additional previous medications included both atorvastatin 20 mg and losartan 50 mg daily.

Upon arrival to the ED, the patient’s vital signs included: heart rate 85, blood pressure 130/85 mmHg, temperature 36.6°C, and room air oxygenation 100%. The patient was overweight but not morbidly obese. Physical examination was remarkable for diffuse abdominal tenderness but most pronounced in the upper abdomen without rebound or guarding. The remainder of the physical examination was unremarkable. Electrocardiogram revealed a normal sinus rhythm without ST segment changes. Initial laboratory evaluation revealed a normal white blood cell count, hyponatremia, hypochloremia, decreased bicarbonate, anion gap acidosis, normal glucose, and normal lipase (Table 1). Liver function testing (LFT) was ordered but laboratory personnel reported hemolysis and requested a redraw of blood samples on three separate occasions. 

**Table 1:** Laboratory values ED: emergency department; AST: aspartate aminotransferase; ALT: alanine aminotransferase

Laboratory Value Name	Reference Range	Date Collected		
		Day 1 (in ED)	Hospital Day 2	Hospital Day 3
White Blood Cell Count	3.4 - 9.6 x 10^9^/L	4.9 x 10^9^/L	4.3 x 10^9^/L	3.4 x 10^9^/L
Sodium	135 -145 mmol/L	128 mmol/L	133 mmol/L	135 mmol/L
Chloride	98 - 107 mmol/L	93 mmol/L	97 mmol/L	101 mmol/L
Bicarbonate	22 - 29 mmol/L	18 mmol/L	21 mmol/L	22 mmol/L
Anion Gap	7 - 15	17	15	12
Glucose	70 -140 mg/dL	126 mg/dL	88 mg/dL	78 mg/dL
Triglycerides	Normal < 150 mg/dL	2562 mg/dL	1381 mg/dL	405 mg/dL
Cholesterol	Normal < 200 mg/dL	539 mg/dL	470 mg/dL	466 mg/dL
AST	8 - 48 U/L	376 U/L	333 U/L	287 U/L
ALT	7 - 15 U/L	140 U/L	129 U/L	103 U/L
Alkaline Phosphatase	40 - 129 U/L	363 U/L	307 U/L	253 U/L
Total Bilirubin	0 - 1.2 mg/dL	1.3 mg/dL	1.4 mg/dL	1.3 mg/dL

**Figure 1:**
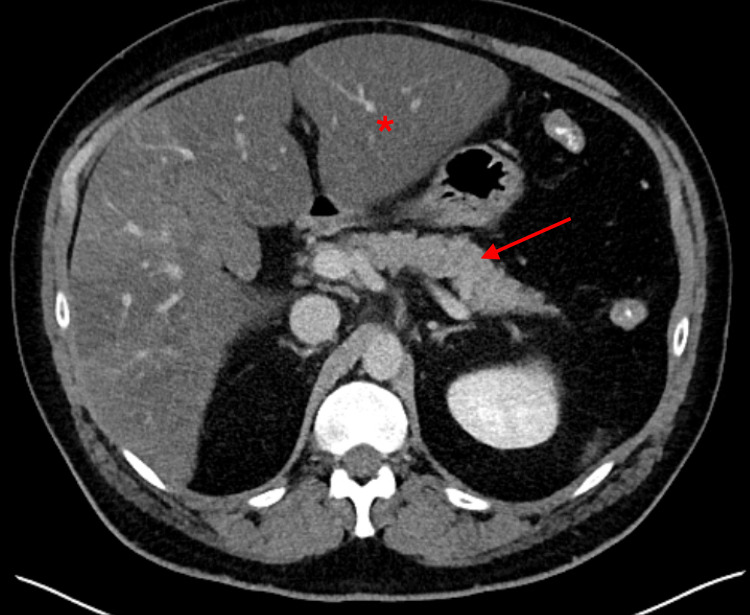
Initial CT revealing liver hypoattenuation consistent with hepatic steatosis (asterisk) and normal appearance of pancreas (arrow)

In the meantime, given the patient’s findings on physical examination, computed tomography (CT) of the abdomen and pelvis with intravenous (IV) contrast was performed. CT imaging revealed no signs of pancreatitis but hepatic steatosis with morphologic change of liver disease was noted (Figure 1). There were no other CT findings to explain the patient’s abdominal discomfort. 

**Figure 2:**
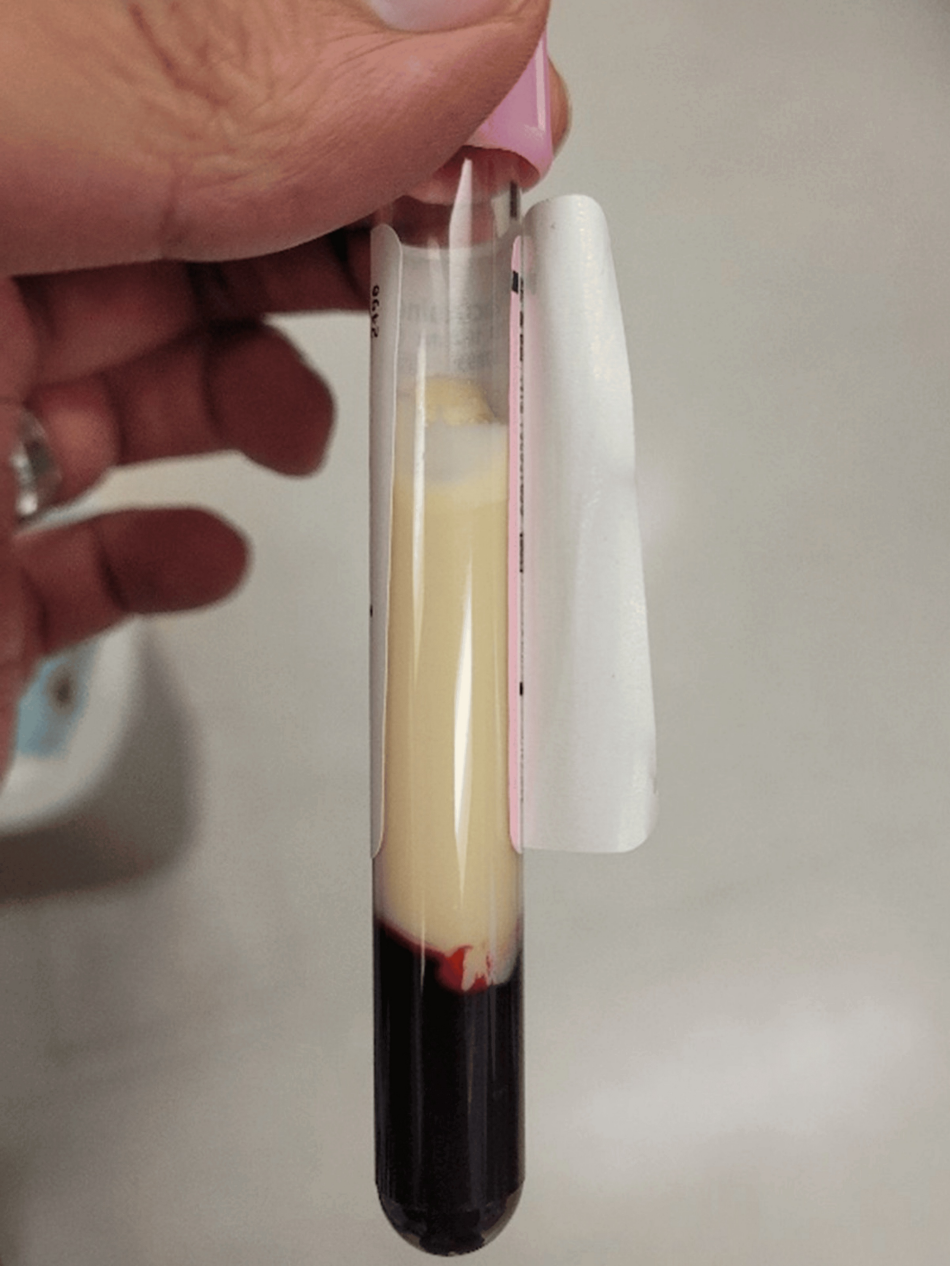
Blood sample with gross lipemia after centrifugation

After further discussion with the laboratory personnel, LFT results appeared to be delayed due to lipemic blood samples (Figure 2). This prompted the evaluation of the patient’s lipid panel in the ED. The patient’s triglycerides were critically elevated to 2562 mg/dL consistent with very severe hypertriglyceridemia, and total cholesterol was also moderately elevated to 539 mg/dL (Table 1). Just two months prior to the ED encounter, the patient had outpatient labs that showed triglycerides of 497 mg/dL and total cholesterol of 205 mg/dL.

Given this constellation of laboratory findings, the Medicine team was consulted for treatment and admission for likely HLAC. In the ED, the patient was kept on nothing by mouth with administration of IV fluids and IV ondansetron 4 mg for ongoing complaints of nausea. Soon after admission, the Endocrinology service was consulted and recommended initiation of IV insulin infusion starting at 0.1-0.3 units/kg/hour with maintenance dextrose containing IV fluids to keep blood glucose stable. The Endocrinology team advised continuation of the IV insulin infusion until triglyceride levels were below 500 mg/dL.

LFTs collected later showed mild transaminitis with elevation of aspartate aminotransferase (AST), alanine aminotransferase (ALT), alkaline phosphatase, and total bilirubin (Table 1). Ultrasound imaging of the abdomen was also performed, and this was unremarkable other than hepatic steatosis. The insulin infusion was continued alongside dextrose 5% in water IV fluids at 150 mL/hour. There was one isolated episode of mild hypoglycemia to 61 mg/dL requiring a decrease in insulin infusion dosing, but otherwise, the patient tolerated the treatment well. There was a corresponding decrease in triglycerides over the course of the patient’s hospitalization from the initial very severe high levels (Table 1). Given this response, plasmapheresis was deemed unnecessary.

No family history of severe hypertriglyceridemia was uncovered during the hospital course. The Endocrinology service suspected that the patient’s dietary habits and alcohol use likely contributed to the ED presentation of HLAC. On hospital day 3, triglycerides decreased from 531 mg/dL in the morning to 405 mg/dL by the latter portion of the day. Upon recommendations of the Endocrinology team, he was discharged home and prescribed Icosapent ethyl at 2 g orally daily with escalation of his statin therapy as tolerated. If a lack of response was seen with these recommendations in the outpatient setting, the Endocrinologist also suggested the addition of fenofibrate. In a subsequent follow-up two weeks later in the clinic setting, the patient was tolerating Icosapent ethyl with a good response in triglyceride levels to 111 mg/dL.

## Discussion

HLAC is a critical condition characterized by severe hypertriglyceridemia, often leading to abdominal pain and potential complications such as pancreatitis [5]. The pathophysiology involves the accumulation of triglyceride-rich lipoproteins, which can cause abdominal symptoms due to visceral organ involvement or hyperviscosity effects [6]. The acute management of HLAC focuses on rapidly lowering triglyceride levels to prevent complications. While insulin infusion is commonly employed, plasmapheresis may be indicated in specific scenarios, particularly when patients present with severe symptoms or pancreatitis. In our patient’s case, without evidence of acute pancreatitis on laboratory evaluation or imaging, insulin infusion was the preferred first-line therapy.

Insulin plays a pivotal role in managing severe hypertriglyceridemia by activating lipoprotein lipase (LPL), which facilitates the breakdown of triglycerides into free fatty acids. This mechanism accelerates the clearance of chylomicrons from the bloodstream, effectively lowering triglyceride levels [7]. Insulin therapy is especially beneficial for patients with concurrent hyperglycemia and hypertriglyceridemia, as it addresses both conditions simultaneously [8,9]. For example, in a recent case report of a patient with poorly controlled insulin-requiring diabetes mellitus, intravenous insulin infusion alone effectively lowered triglyceride levels from greater than 11,000 mg/dL to less than 1000 mg/dL over a four-day hospitalization [10].

Plasmapheresis is a more invasive therapeutic procedure that removes triglyceride-rich lipoproteins from circulation, providing a rapid means to lower triglyceride levels [9]. It is particularly indicated in cases where patients exhibit severe symptoms, such as pancreatitis or hyperviscosity syndrome, which may not respond adequately to insulin therapy alone [9]. A small case series reported an 80% reduction in plasma triglycerides following plasma exchange in patients with hypertriglyceridemia-induced pancreatitis, demonstrating its quick efficacy in acute settings [11].

However, when comparing the two main treatment options for HLAC, insulin infusion is increasingly preferred over plasmapheresis for managing severe hypertriglyceridemia, particularly in the context of hypertriglyceridemic pancreatitis [12]. While plasmapheresis can rapidly lower triglyceride levels, some studies suggest that insulin infusion may lead to better clinical outcomes. For example, one randomized controlled trial indicated that although plasmapheresis cleared triglycerides more quickly, patients treated with insulin had a lower incidence of progression to severe pancreatitis and persistent organ failure [13]. This observation may be due to insulin not only reducing triglyceride synthesis but also accelerating the metabolism of free fatty acids thought to be more directly implicated in pancreatic injury [14]. Consequently, insulin's multifaceted mechanism of action can mitigate the risk of pancreatitis more effectively than plasmapheresis, despite the latter's rapid triglyceride clearance [14]. Additionally, insulin infusion is less invasive, easier to initiate, and associated with fewer complications compared to plasmapheresis, which requires hemodialysis access and carries risks such as bleeding and infection [15,16]. Furthermore, while plasmapheresis may lower triglyceride levels faster than an insulin infusion, plasmapheresis does not show long-term benefits of reduced mortality or duration of hospital stay [4]. Thus, in acute settings such as the ED where rapid intervention is critical, insulin infusion emerges as a safer and more clinically beneficial option for managing hypertriglyceridemia, as was observed in our patient’s case.

Few case reports exist documenting cases of severe hypertriglyceridemia without pancreatitis but still requiring intervention and treatment. In one case, an asymptomatic patient had triglyceride levels exceeding 11,000 mg/dL associated with acute liver dysfunction, necessitating management with insulin infusion [17]. This underscores the importance of treatment even in asymptomatic cases to prevent progression to complications including pancreatitis. For example, another case report of a patient with severe hypertriglyceridemia and acute pancreatitis highlights the progression to portal vein thrombosis and intra-abdominal hypertension; prompt management with insulin therapy in this case was successful [18]. Despite the usual response of insulin infusion in treatment of severe hyperlipidemia, another report emphasizes that despite aggressive management with insulin infusion, patients may require an extended hospitalization to achieve acceptable triglyceride levels [19]. That particular case illustrates the variability in patient responses to insulin and highlights the need for alternative therapies, such as plasmapheresis, when insulin is insufficient [19].

Of note, in our patient’s case, the finding of lipemia caused a delay in LFT results. This was a clue that prompted further evaluation with a lipid panel, which is not routinely obtained in the ED setting. Lipemia can significantly interfere with laboratory testing, leading to erroneous results. The presence of high levels of triglycerides can cause turbidity in serum samples, which may affect spectrophotometric measurements and lead to inaccurate reporting of various laboratory parameters, including glucose and electrolyte levels [2,20]. This interference underscores the importance of recognizing lipemia during laboratory evaluation and potentially re-evaluating samples after addressing the underlying hyperlipidemia.

Our case is particularly noteworthy due to the primary presenting complaint of abdominal pain without evidence of pancreatitis on initial evaluation. Many cases of HLAC are accompanied by acute pancreatitis; however, the current patient’s presentation highlights that HLAC can manifest solely as abdominal discomfort. This emphasizes the need for clinicians to maintain a high index of suspicion for hyperlipidemia-related complications even when traditional signs of pancreatitis are absent.

## Conclusions

HLAC represents a diagnostic challenge requiring prompt recognition and intervention. Understanding the mechanisms behind treatment modalities like insulin infusions and plasmapheresis are essential for effective management. Additionally, awareness of laboratory interference due to lipemia is crucial for accurate diagnostic assessment. Laboratory personnel can be pivotal by alerting the provider of this finding during their analysis of blood samples. No traditional familial or genetic risk factors were uncovered in our patient, suggesting that dietary habits and alcohol consumption were secondary causes for his severe hypertriglyceridemia. This case illustrates the importance of considering HLAC in atypical presentations and reinforces the need for tailored treatment strategies to mitigate potential complications.
